# Equivalence classes of circular codes induced by permutation groups

**DOI:** 10.1007/s12064-020-00337-z

**Published:** 2021-02-01

**Authors:** Fariba Fayazi, Elena Fimmel, Lutz Strüngmann

**Affiliations:** 1 Department of Mathematics, Faculty of Science, University of Qom, Qom, IR Iran; 2 Faculty of Computer Science, Insitute for Mathematical Biology, Mannheim University of Applied Science, Mannheim, Germany

**Keywords:** Circular codes, Comma-free codes, Symmetric group, Frame retrieval, Translation, Signal processing, Symmetric group

## Abstract

In the 1950s, Crick proposed the concept of so-called comma-free codes as an answer to the frame-shift problem that biologists have encountered when studying the process of translating a sequence of nucleotide bases into a protein. A little later it turned out that this proposal unfortunately does not correspond to biological reality. However, in the mid-90s, a weaker version of comma-free codes, so-called circular codes, was discovered in nature in J Theor Biol 182:45–58, 1996. Circular codes allow to retrieve the reading frame during the translational process in the ribosome and surprisingly the circular code discovered in nature is even circular in all three possible reading-frames ($C^3$-property). Moreover, it is maximal in the sense that it contains 20 codons and is self-complementary which means that it consists of pairs of codons and corresponding anticodons. In further investigations, it was found that there are exactly 216 codes that have the same strong properties as the originally found code from J Theor Biol 182:45–58. Using an algebraic approach, it was shown in J Math Biol, 2004 that the class of 216 maximal self-complementary $C^3$-codes can be partitioned into 27 equally sized equivalence classes by the action of a transformation group $L \subseteq S_4$ which is isomorphic to the dihedral group. Here, we extend the above findings to circular codes over a finite alphabet of even cardinality $|\Sigma |=2n$ for $n \in {\mathbb {N}}$. We describe the corresponding group $L_n$ using matrices and we investigate what classes of circular codes are split into equally sized equivalence classes under the natural equivalence relation induced by $L_n$. Surprisingly, this is not always the case. All results and constructions are illustrated by examples.

## Introduction

Crick et al. (1957) proposed a class of trinucleotide codes—called comma-free codes—as nature’s key to avoid errors when translating the genetic code. In Crick’s biological setting, comma-free codes used a subset of the 64 possible codons for coding the 20 amino acids in a way such that they allowed the detection of errors in the translation process from coding sequences to proteins. Thus, these codes did not only raise interest from biologists but also were of great interest from the point of coding theory because they form a particular type of error correcting codes. Naturally, combinatorial properties of comma-free codes were studied extensively thereafter passing from the biological setting to words of arbitrary fixed length over alphabets of arbitrary size (see Golomb et al. 1958a and Golomb et al. 1958b). A series of papers was inspired by these seminal works, mostly dealing with purely combinatorial aspects of comma-free codes and finally posing some challenging open problems (see Cummings 1976; Eastman 1965; Levenshtein 2004; Scholtz 1969; Tang et al. 1987; Ball and Cummings 1976b; Bilotta et al. 2013). Later on, also strong comma-free codes (under the name of strongly regular codes or non-overlapping codes) were investigated and gained interest in automata theory as well as the theory of frame synchronization (see Blackburn 2015; Levenšteĭn 1964, 1970 as well as Bajić and Stojanović 2004; Bilotta et al. 2013, 2012; Chee et al. 2013; Guibas and Odlyzko 1978).

However, in the early 1960s, after the Poly-U experiment by Nirenberg and Matthaei, it became clear that the proposal of Crick appraise to be wrong (Hayes 1998). In fact, there are 408 maximal comma-free codes (Golomb et al. 1958a) that code for at most 13 amino acids (Michel 2014). Nevertheless, recent works have shown that instead of comma-free codes, a weaker class of codes—called circular codes—is indeed used in protein-coding sequences. Circular codes are a less confined version of comma-free codes and can be used for normal reading frame retrieval (se Arquès and Michel 1996; Michel et al. 2008; Michel 2020). A particular circular code—called *X*—had been found by extensive statistical investigations in large samples of genetic data of archaea, plasmids and viruses, in addition to bacteria and eukaryotes (see Arquès and Michel 1996; Michel 2015, 2017). The code *X* contains the following 20 trinucleotides:

$$\begin{aligned} X= & {} \{AAC,AAT,ACC,ATC,ATT, CAG,CTC,CTG,GAA,GAC,\\&GAG,GAT,GCC,GGC,GGT,GTA, GTC,GTT,TAC,TTC\}. \end{aligned}$$

Arquès and Michel did not only discover that this code was able to detect frame-shifts in the normal reading frame but also in the two shifted frames and it is self-complementary which means that it is symmetric with respect to the double helix structure of the DNA. In Arquès and Michel (1996), it is proved that there exist exactly 216 such codes—called maximal self-complementary $C^3$-codes. Among these 216 codes, the maximal number of amino acids that can be coded is 14 (see Michel 2014) while comma-free codes which are self-complementary, or $C^3$, or $C^3$ self-complementary can contain at most 16 trinucleotides and code for at most 11 amino acids (Michel 2020).

Fimmel et al. (2014) later showed that the class of 216 maximal self-complementary $C^3$-codes over the genetic four-letter alphabet $\Sigma =\{A,C,G,T\}$ can be partitioned into 27 equal-sized classes so that each of these equivalence classes has eight maximal self-complementary $C^3$-codes that are related by a subset of transformations $L \subseteq S_{\Sigma }$ of the symmetric group $S_{\Sigma }$. The use of the symmetric group in order to study circular codes had already been initiated in Michel and Pirillo (2011). The transformations in *L* are exactly those permutations of $\Sigma$ that preserve self-complementarity and it was shown that *L* is isomorphic to the Dihedral group of order 8. The important implication of this result is that all codes in one equivalence class share the same error detecting properties while those in different classes are stronger or weaker. Moreover, recent findings by Seligman and others show that applying a systematic change of bases to RNA, e.g. by applying a transformation from $S_{\{A,C,G,T\}}$, may lead to existing RNA—called Swinger RNA (Seligman 2016; Michel and Seligmann 2014). Particularly, the transformations from *L* turned out to yield such Swinger RNA. It is tempting to speculated that nature may use this mechanism in order to encode not only one set of information in DNA but 8 (the size of *L*) or even 24 (the size of $S_{\{A,C,G,T\}}$) sets at the same time.

In the present work, we extend the previous results to a finite alphabet of even length $|\Sigma |=2n$ for $n \in {\mathbb {N}}$ and generalize the group *L* to $L_n$. The motivation to restrict ourselves to alphabets of even cardinality obviously comes from biology due to the sets of complementary bases there. However, the presented approach could also be investigated for alphabets of odd size but some of the constructions, e.g. in Theorem 4.4, would not work in that case. In  “ Generalization of the group *L*” section, we describe $L_n$ and some of its properties using matrices. In “Equivalence classes of codes induced by the action of the group L_n_” section , we discuss equivalence classes of codes with respect to the action of the group $L_n$. In general, we can’t reach equally sized classes for any class of codes. For instance, we will show that maximal self-complementary circular codes can’t be classified into equally sized equivalence classes due to the action of $L_n$ (see Example 4.18). On the other hand, we prove that dinucleotide circular codes are divided into equally sized equivalence classes due to the action of $L_n$ (see Lemma 4.19) and the same holds true for the general class of $\mu$-maximum circular $C^l$-codes over general alphabets (see Theorem 4.20).

## Definitions and Notions

Let $\Sigma$ be an arbitrary finite alphabet of size *m*. For a natural number $l \ge 2$ an *l*-*letter code* simply is a subset $X \subseteq \Sigma ^l$ where $\Sigma ^l$ is the set of all words of length *l* over $\Sigma$ (the length of a word is the number of its letters, e.g. $x_1 \cdots x_n$ has length *n*) . As usual, $\Sigma ^*$ denotes the set of all finite length words over $\Sigma$, i.e. $\Sigma ^*=\bigcup \nolimits _{n \in {{\mathbb {N}}}}\Sigma ^n$ including the empty word $\epsilon$. Given $v,w \in \Sigma ^*$, we call *v* a *prefix* of *w* if $w=vv'$ for some $v' \in \Sigma ^*$ and we call *v* a *suffix* of *w* if $w=v'v$ for some $v' \in \Sigma ^*$. Moreover, if $w=x_1 \cdots x_n \in \Sigma ^n$, then $\alpha _i(w)=x_{i+1} \cdots x_n \cdot x_1 \cdots x_i$ is called the *i-th circular shift of* *w* for $n-1 \ge i \ge 1$ and we put $\alpha _0(w)=w$. This notion obviously extends to sets, i.e. $\alpha _i(X)=\{ \alpha _i(w)| w \in X \}$ for a set $X \subseteq \Sigma ^n$.

We recall a few classical definitions of codes as follows:

### Definition 2.1

Let $X \subseteq \Sigma ^ l$ be an *l*-letter code and $k \in {\mathbb {N}}$. We say that *X* is

1. a *k-circular* *l-letter code* if for any $m \le k$ and any concatenation $x_1 \cdots x_m$ of *l*-tuple from *X* there is only one partition into *l*-tuple from *X* when read on a circle. In other words, for any $1 \le i \le l-1$ the circular shift $\alpha _i(x_1 \cdots x_m) \not \in X^m$;
2. a *circular l-letter code* if it is a *k*-circular *l*-letter code for all $k \in {\mathbb {N}}$;
3. a *strong comma-free code* if no $v \in \Sigma ^*$, $v \not = \epsilon$ appears both as a prefix and a suffix in *X*. In other words, given any two non-necessarily distinct elements $b_1 = x_1 \cdots x_l$ and $b_2 = y_1 \cdots y_l$ of *X*, for every $k \in \{1, . . . , l - 1\}$ we have
  $$\begin{aligned} x_{l+1-k } \cdots x_l \ne y_1 \cdots y_k; \end{aligned}$$
4. a *comma-free code* if for any two elements $x_1 \cdots x_l$ and $y_1 \cdots y_l$ in *X*, we have
  $$\begin{aligned} \forall i \in \{2, . . . , l\} \quad x_i \cdots x_ly_1 \cdots y_{i-1} \not \in X; \end{aligned}$$
5. a *maximal* $(k-)$*circular (comma-free, strong comma-free)* *l*-*letter code* if it is not contained in a larger $(k-)$circular code;
6. a *maximum* $(k-)$ *circular (comma-free, strong comma-free)* *l*-*letter code* or, equivalently, code of maximal size if $|Y | \le |X|$ whenever *Y* is a $(k-)$circular (comma-free, strong comma-free) *l*-letter code over $\Sigma$.

Obviously, any strong comma-free code is also comma-free and hence also circular. Moreover, a maximum code is certainly also maximal. There is a general upper bound for the size of a maximum *l*-letter code ((*k*-)circular, comma-free, strong comma-free), namely the maximal size of a 1-circular *l*-letter code over $\Sigma$. Such a code can contain at most one element from each of the *complete* classes $\{\alpha _i(x_1 \cdots x_l) \mid i \le l \}$ with $x_1 \cdots x_l \in \Sigma ^l$. Here complete means that the size of this set is equal to *l*. The number of such complete classes is given by

$$\begin{aligned} \frac{1}{\ell } \sum _{d | \ell } \mu \left( \frac{\ell }{d}\right) m^d, \end{aligned}$$

where $m = |\Sigma |$ and $\mu$ is the Möbius function and it was shown in Fimmel et al. (2019) that all maximum circular *l*-letter codes over $\Sigma$ indeed have this size. However, this is not known for other classes of codes and therefore we add the following definition.

### Definition 2.2

Let $X \subseteq \Sigma ^ l$ be an *l*-letter code. We say that *X* is $\mu$-maximum if its size is equal to $\frac{1}{\ell } \sum _{d | \ell } \mu \left( \frac{\ell }{d}\right) m^d$.

An interesting class of codes is also given by the so-called $C^n$-codes. Note that it is not known if a maximum $C^n$-code is also $\mu$-maximum.

### Definition 2.3

A circular code $X \subseteq \Sigma ^n$ is called a $C^n$-code if also $\alpha _i(X)$ is circular for all $1 \le i \le n-1$. In other words, the shifted codes of *X* are also circular.

For the convenience of the reader, we give some examples in a biological setting choosing $\Sigma ={\mathcal {B}}=\{ A,C,T,G\}$ to be the genetic alphabet. Here, *A* stands for adenine, *C* stands for cytosine, *G* stands for guanine and *T* stands for thymine (see Fimmel and Strüngmann 2018 for more details).

### Example 2.4

We have the following for ${\mathcal {B}}=\{ A,C,G,T \}$.

1. Let $X=\{ AAC,AAT,ACC,ATC,ATT,CAG,CTC,CTG,GAA,GAC, GAG,GAT,$ $GCC,GGC,GGT,GTA,GTC,GTT,TAC,TTC \}$. Then, $X \subseteq \Sigma ^3$ is a maximum (and hence maximal) circular triletter $C^3$-code.
2. Let $X=\{ CGT,ACG,TAC,GTA \}$. Then, $X \subseteq \Sigma ^3$ is a 3-circular code but not a 4-circular triletter code.
3. Let $X=\{AAC, AGC, AT C, GAC, GGC, GT C, T AC, T GC, T T C\}$. Then, $X\subseteq \Sigma ^3$ is a maximum (and hence maximal) strong comma-free triletter code.

We will also consider the so-called *symmetric group* acting on the elements of the alphabet $\Sigma$ which is defined as

$$\begin{aligned} S_{\Sigma }:=\{\pi : \Sigma \rightarrow \Sigma | \pi ~is~ bijective~\} \end{aligned}$$

endowed with the usual group operation given by the composition of functions. The group $S_{\Sigma }$ has $| \Sigma | !$ elements and for every $l \in {\mathbb {N}}$, any bijective mapping $\pi : \Sigma \rightarrow \Sigma$ can be applied componentwise to $x \in \Sigma ^l$ and thus induces a bijective map $\Sigma ^l \rightarrow \Sigma ^l$, which is also called $\pi$. A bijection $\pi$ of $S_{\Sigma }$ is an *involutory function (or an involution)* if $\pi \circ \pi (x) = x$ for every $x \in \Sigma$, i.e. $\pi$ is of order 2. A *fixed point* of a bijection $\pi \in S_{\Sigma }$ is an element $x \in \Sigma$ such that $\pi (x) = x$. We will state a remark here that is clear but important in the sequel of the paper since it justifies why we will restrict to alphabets of even cardinality in the next section.

### Remark 2.5

If the cardinality of $|\Sigma |$ is even, then $S_{\Sigma }$ contains involutory bijections without fixed points.

We will need some further notations from general group theory (see Hall 1970; Rotman 1995 for further details). Give a group *G* and a subset $S \subseteq G$ of *G*, the *centralizer* $C_G(S)$ of *S* in *G* is the set of all elements of *G* that commute with all elements of *S*, i.e. $C_G(S)=\{ g \in G | g \circ s = s \circ g {\textit{ for all }} s \in S \}$. Moreover, the *normalizer* of *S* in *G* is the set of all elements *g* of *G* that commute with *S* as a set but not necessarily point wise, i.e. $N_G(S)=\{ g \in G| g\circ S= S \circ g \}$. Both, centralizer and normalizer are subgroups of the group.

Motivated by recent results in mathematical biology related to the genetic code, we also define $\pi$-*self-complementarity* of a code for some involution $\pi \in S_{\Sigma }$. Fix such an involution $\pi$ and a code $X \subseteq \Sigma ^n$, then *X* is called $\pi$-*self-complementary* if $\overset{\leftarrow }{\pi (X)}=X$. Here, $\overset{\leftarrow }{}$ is the reversing operation and assigns to a word $w=x_1 \cdots x_n \in \Sigma ^n$ the reversed word $x_n \cdots x_1$.

Again for the convenience of the reader, we give an example in the biological setting for ${\mathcal {B}}=\{A,C,G,T\}$ the genetic alphabet. Fix *c* as the permutation $\pi _{(AT)(CG)}$ that switches *C* and *G* and *A* and *T*, it is easy to see that the code

$$\begin{aligned} X= & {} \{AAC,AAT,ACC,ATC,ATT,CAG, CTC,CTG,GAA,GAC,\\&GAG,GAT,GCC,GGC,GGT,GTA, GTC,GTT,TAC,TTC\}. \end{aligned}$$

is a *c*-self-complementary circular code.

We conclude this section with an easy result that shows that circularity, (strong)-comma freeness and also the $C^n$-property of codes are preserved under permutations of the alphabet $\Sigma$, i.e. under the action of $S_{\Sigma }$.

### Proposition 2.6

*Let* $\Sigma$ *be a finite alphabet and* $X \subseteq \Sigma ^n$ *a circular (respectively, comma-free, strong comma-free*, $C^n$-) *code. If* $\pi \in S_{\Sigma }$, *then* $\pi (X)$ *is again a circular (respectively, comma-free, strong comma-free*, $C^n$-) *code*.

### Proof

Easy—see also (Fimmel and Strüngmann 2018). $\square$

However, in contrast to the above proposition, it is not true in general that permutations from $S_{\Sigma }$ preserve $\pi$-self-complementarity of codes. In fact, it was shown in Fimmel et al. (2014) that in the setting of the genetic code, there are exactly eight permutations that preserve *c*-self-complementarity with *c* from above.

### Proposition 2.7

*Let* $\Sigma ={\mathcal {B}}=\{A,C,G,T\}$ *be the genetic alphabet and* $c=\pi _{(AT)(CG)}$. *A permutation* $\pi \in S_{\Sigma }$ *preserves* *c*-*self-complementarity of all circular codes* $X \subseteq \Sigma ^3$ *if and only if* $\pi$ *commutes with* *c*.

### Proof

For the proof see (Fimmel et al. 2014). $\square$

In Fimmel et al. (2014), it turned out that the only permutations from $S_{\Sigma }$ (with $\Sigma ={\mathcal {B}}=\{A,C,G,T\}$) that commute with $c=\pi _{(AT)(CG)}$ are the following eight permutations that form a subgroup *L* of the symmetric group $S_{\Sigma }$ which is isomorphic to the dihedral group $D_4$ - the symmetry group of the square:

+ $$\begin{aligned} L&:=\{id, \pi _{{(CG)}{(AT)}}(=c), \pi _{{(CT)}{(AG)}}(=p), \\&\quad \pi _{{(CA)}{(GT)}}(=r),\pi _{{(CG)}}, \pi _{{(AT)}} \pi _{{(ACTG)}}, \pi _{{(AGTC)}}\}. \end{aligned}$$

This group will be studied later on in more detail and its multiplication table is given in Table 1:

**Table 1:** Cayley table of the group *L*

$\circ$	*e*	*c*	*p*	*r*	$\pi _{(AT)}$	$\pi _{(CG)}$	$\pi _{(ACTG)}$	$\pi _{(AGTC)}$
*e*	*e*	*c*	*p*	*r*	$\pi _{(AT)}$	$\pi _{(CG)}$	$\pi _{(ACTG)}$	$\pi _{(AGTC)}$
*c*	*c*	*e*	*r*	*p*	$\pi _{(CG)}$	$\pi _{(AT)}$	$\pi _{(AGTC)}$	$\pi _{(ACTG)}$
*p*	*p*	*r*	*e*	*c*	$\pi _{(AGTC)}$	$\pi _{(ACTG)}$	$\pi _{(AT)}$	$\pi _{(CG)}$
*r*	*r*	*p*	*c*	*e*	$\pi _{(ACTG)}$	$\pi _{(AGTC)}$	$\pi _{(CG)}$	$\pi _{(AT)}$
$\pi _{(AT)}$	$\pi _{(AT)}$	$\pi _{(CG)}$	$\pi _{(ACTG)}$	$\pi _{(AGTC)}$	*e*	*c*	*r*	*p*
$\pi _{(CG)}$	$\pi _{(CG)}$	$\pi _{(AT)}$	$\pi _{(AGTC)}$	$\pi _{(ACTG)}$	*c*	*e*	*p*	*r*
$\pi _{(ACTG)}$	$\pi _{(ACTG)}$	$\pi _{(AGTC)}$	$\pi _{(AT)}$	$\pi _{(CG)}$	*r*	*p*	*c*	*e*
$\pi _{(AGTC)}$	$\pi _{(AGTC)}$	$\pi _{(ACTG)}$	$\pi _{(CG)}$	$\pi _{(AT)}$	*p*	*r*	*e*	*c*

Consequently, the group *L* acts on the class of *c*-self-complementary codes. In the sequel of the paper, we will generalize this result to arbitrary alphabets and circular codes.

## Generalization of the group *L*

In Fimmel et al. (2014), Fimmel *et* *al* showed that the class of 216 maximal *c*-self-complementary $C^3$-codes over $\Sigma ={\mathcal {B}}=\{A,C,G,T\}$ can be partitioned into 27 equal-sized equivalence classes under the action of the subgroup *L* of the symmetrical group from (+). This representation had many implications on the study of these codes since the codes from the same equivalence class have the same error-detecting properties (Fimmel et al. 2014) and also share other properties. We now intend to generalize this approach and first define the group *L* in the more general setting. Let $\Sigma$ be a finite alphabet as before.

### Lemma 3.1

*Let* $S_{\Sigma }$ *be the symmetric group acting on the elements of the alphabet* $\Sigma$ *and let* $\alpha \in S_{\Sigma }$ *be a permutation. Moreover, let* $L_{\Sigma }^{\alpha } \subseteq S_{\Sigma }$ *be the set of all bijections which commute with* $\alpha$, *i.e*.

$$\begin{aligned} L_{\Sigma }^{\alpha }=\{\pi \in S_{\Sigma }| \pi \circ \alpha =\alpha \circ \pi \} \end{aligned}$$

*Then,* $L_{\Sigma }^{\alpha }$ *is a subgroup of* $S_{\Sigma }.$

### Proof

Clearly, $L_{\Sigma }^{\alpha }$ is the centralizer of $\alpha$ in $S_{\Sigma }$ and hence a subgroup of $S_{\Sigma }$ as it is well known in group theory (see, e.g. Hall 1970). $\square$

We now want to determine the structure of $L_{\Sigma }^{\alpha }$ when $\alpha$ is an involution without fixed points. Thus, we will be assuming from now on and for the rest of the paper that $\Sigma$ will denote an alphabet of even cardinality with $|\Sigma | = 2n$ for some $n\in {{\mathbb {N}}}$. Moreover, we will assume that $c \in S_{\Sigma }$ is an involutory bijection without fixed points and we abbreviate $L_{\Sigma }^c$ by $L_n=L_{\Sigma }^{c}$.

### Description of $L_n$ using matrices

In this subsection we first develop a description of the group $L_n$ using matrices. Recall that $S_{\Sigma }$ is the symmetric group acting on the elements of the alphabet $\Sigma$ where $|\Sigma | = 2n$ is even and $c \in S_{\Sigma }$ is an involution without fixed points. Moreover, $L_n=\{\pi \in S_{\Sigma }| \pi \circ c=c \circ \pi \}$ is the centralizer of *c* in $S_{\Sigma }$.

Let $\Delta$ be the ring of all $2 \times 2$-matrices over the field $F_2=\{0,1\}$ with two elements.

#### Lemma 3.2

*The subgroup* $L_n \subseteq S_{\Sigma }$ *of all bijections which commute with* *c* *is isomorphic to the group of all* $n \times n$- *matrices over* $\Delta$ *such that in each row and in each column there is exactly one non-trivial* $\delta \in \Delta$ *of the form* $$\bigl ( {\begin{matrix} 1&{}0\\ 0&{}1 \end{matrix}} \bigr )$$ or $$\bigl ( {\begin{matrix} 0&{}1\\ 1&{}0 \end{matrix}} \bigr )$$. *Thus, we have* $$\begin{aligned} |L_n|=n! 2^n. \end{aligned}$$

#### Proof

Without loss of generality we may assume that

$$\begin{aligned} \Sigma = \{1, 2, \ldots , 2n\} {\textit{ and }} c = (12)(34)(56)\ldots ((2n- 1)2n). \end{aligned}$$

Clearly, any $\pi \in S_{\Sigma }$ can be represented as a $(2n \times 2n)$-matrix with binary entries such that in every column and in every row there is exactly one entry equal to 1 and the remaining entries are 0. In this representation, *c* has the following form:

$$\begin{aligned}\begin{pmatrix} 0&{}1&{}0&{}0&{}...&{}0 \\ 1&{}0&{}0&{}0&{}...&{}0 \\ 0&{}0&{}0&{}1&{}...&{}0 \\ 0&{}0&{}1&{}0&{}...&{}0 \\ ...&{}...&{}...&{}...&{}...&{}... \\ 0&{}0&{}0&{}0&{}...&{}1 \\ 0&{}0&{}0&{}...&{}1&{}0 \end{pmatrix}\end{aligned}$$

Now, the product $c \circ \pi$ is obtained from the matrix associated to $\pi$ by swapping the $(2i-1)$th and the (2*i*)th rows for $(i = 1,\ldots , n)$, while the product $\pi \circ c$ is obtained from the matrix associated to $\pi$ by swapping the $(2j - 1)$th and the (2*j*)th columns for $(j = 1,\ldots , n)$. If $c \circ \pi =\pi \circ c$, then we thus have for every pair $i, j = 1,\ldots , n$

$$\begin{aligned} \begin{pmatrix} a_{(2i-1)(2j)}&{}a_{(2i-1)(2j-1)}\\ a_{(2i)(2j)}&{}a_{(2i)(2j-1)} \end{pmatrix}= \begin{pmatrix} a_{(2i)(2j-1)}&{}a_{(2i)(2j)}\\ a_{(2i-1)(2j-1)}&{}a_{(2i-1)(2j)} \end{pmatrix} \end{aligned}$$

There are only three $2 \times 2$-matrices with binary entries having at most one 1 in every row and column fulfilling this condition:

$$\begin{aligned}E_1= \bigl ( {\begin{matrix} 1&{}0\\ 0&{}1 \end{matrix}} \bigr ), E_2= \bigl ( {\begin{matrix} 0&{}1\\ 1&{}0 \end{matrix}} \bigr ), {\textit{ and }}E_3= \bigl ( {\begin{matrix} 0&{}0\\ 0&{}0 \end{matrix}} \bigr )\end{aligned}$$

Recalling that $\pi$ is represented by a binary matrix in which there is exactly one 1 in each row and column, it is obvious that the entire $2n \times 2n$- matrix consists of $n \times n$ blocks from $\Delta$ such that exactly *n* of them will have the shape $E_1$ or $E_2$ and the rest are the trivial matrices $E_3.$ Clearly, there are $n! 2^n$ possibilities for distributing these matrices and hence $|L_n|=n! 2^n.$ $\square$

We would like to remark that there are alternative descriptions of the group $L_n$, e.g. using wreath products.

#### Remark 3.3

The group $L_n$, which we described above, has very interesting group-theoretical properties. For instance, it can be proven that

$$\begin{aligned} L_n\cong C_2 \wr _X S_n \end{aligned}$$

where $X=\{1,2,\ldots ,n\}$ and $C_2$ is the cyclic group of order 2 and at the same time

$$\begin{aligned} L_n\cong C_2^n \ltimes _{\alpha } S_n \end{aligned}$$

where $\ltimes _{\alpha }$ denotes the *(outer) semidirect product* with respect to $\alpha$ and $\wr _X$ is the so-called *wreath product*. It is an interesting investigation in itself to get involved with the group. However, this is outside the scope of the article.

## Equivalence classes of codes induced by the action of the group $L_n$

Recall from the previous sections that $\Sigma$ is a finite alphabet of even size 2*n* and $c \in S_{\Sigma }$ is an involution without fix points that was used to define the group $L_n$ consisting of all permutations in $S_{\Sigma }$ that commute with *c*. It was pointed out in “ Definitions and Notions” Section  that in this case, the mappings from $L_n$ preserve all properties of codes given in Definition 2.1 including *c*-self-complementarity. It is thus natural to define an equivalence relation on codes by setting

$$\begin{aligned} X \sim X^{' } \iff {\textit{ there exists }} \pi \in L_n ~~s.t ~~\pi (X)=X^{'} \end{aligned}$$

for codes *X* and $X'$; the corresponding equivalence class of a code *X* will be denoted by [*X*]. (Of course if the codes are not *c*-self-complementary, then one could use the full symmetric group instead of $L_n$ but for now we will restrict to the group $L_n$). In Fimmel et al. (2014), Fimmel *et* *al* considered this equivalence relation for the class of 216 *c*-self-complementary $C^3$-codes over the genetic alphabet ${\mathcal {B}}=\{A,C,G,T\}$ where $c=\pi _{(AC)(GT)}$. It was shown that the 216 maximal *c*-self-complementary $C^3$-codes can be partitioned into 27 equivalence classes under the action of $L_2$ which are all of size 8 - the size of the group $L_2$. However, in general, we cannot expect to get equivalence classes of the same size as was demonstrated in Keller (2014), Lemegne (2015).

In the following, we will consider different classes of codes and determine if the action of $L_n$ divides the class into equivalence classes of the same size or different sizes. As a basis, recall that Fimmel *et* *al*. in Fimmel et al. (2019) calculated the size and number of maximal *l*-letter circular codes, diletter and triletter comma-free codes, maximum self-complementary comma-free triletter codes, *l*- letter strong comma-free codes and maximal strong self-complementary comma-free triletter codes over $\Sigma$.

Let us remark that in the trivial case $l=1$, the code classes of maximal *c*-self-complementary strong comma-free, strong comma-free, *c*-self-complementary comma-free, comma-free, *c*-self-complementary circular and circular codes coincide. In this case, there is only one maximal 1-letter code of each kind, namely $X=\Sigma$ and, thus, its equivalence class consists of one element.

### Equivalence classes of strong comma-free codes

Let us consider the class of maximal (*c*-self-complementary) strong comma-free codes.

We begin with a consideration of the case of the binary alphabet, in which the situation is rather unspectacular:

#### Proposition 4.1

*Let* $\Sigma =\{ 0,1\}$ *be the binary alphabet. Then,* $L_1=S_{\Sigma }$ *and the only non-trivial permutation is* $c: \{ 0,1 \} \rightarrow \{ 0,1 \}$ *that flips* 0 *and* 1. *Moreover, no strong comma-free* *l*-*letter code over* $\Sigma$ *is invariant under* *c*. *So all equivalence classes have size* 2.

#### Proof

Let *X* be a strong comma-free *l*-letter code over $\Sigma$. If $x \in X$, then *x* must start with 0 and end with 1 or vice versa due to strong comma-freeness (follows immediately from the definition). Without loss of generality, assume that *x* starts with 0 and ends with 1. However, if $c(X)=X$, then *X* would also contain *c*(*x*) which starts with 1 and ends with 0—contradiction to strong comma-freeness. $\square$

Before we continue our considerations about equivalence classes, we have to close a gap and count the number of maximal *c*-self-complementary strong comma-free diletter codes over an alphabet of even cardinality:

#### Lemma 4.2

*Let* $\Sigma$ *be a finite alphabet with* $|\Sigma |=2n$ *for some* $n\in {{\mathbb {N}}}$ *and let* $L_n\subset S_{\Sigma }$ *be the centralizer* $C_{S_{\Sigma }}(c)$ *of some involution* *c* *without fixed points. Then, there are* $2^n$ *different maximal (=maximum)* *c*-*self-complementary strong comma-free diletter codes over* $\Sigma$.

#### Proof

In Fimmel et al. (2017b), the structure of a maximal diletter strong comma-free code over an arbitrary alphabet was described. For instance, if an alphabet is of an even cardinality, it is partitioned into two disjoint sets $T^+$ and $T^-$ of equal size so that each diletter from the code begins with a letter from $T^-$ and ends with a letter from $T^+$. In order to construct a *c*-self-complementary code, we have to ensure that for every $x\in \Sigma$ *x* and *c*(*x*) belong to different sets. There are *n* pairs of complementary letters, thus, we have $2^n$ possibilities to constitute $T^-$ and, correspondingly, $T^+$. $\square$

In general, it can be assumed that in the case of maximum strong comma-free codes, the equivalence classes can be smaller than $|L_n|$, as the following example shows:

#### Example 4.3

Let ${\mathcal {B}}=\{A,C,G,T\}$ be the genetic alphabet. There are exactly eight maximal (=maximum) strong comma-free triletter codes of size 9 as follows:

$$\begin{aligned} X_1= & {} \{AAC,AGC,AT C,GAC,GGC, GTC,TAC,TGC,TTC\},\\ X_2= & {} \{AAG,AC G,AT G,CAG,CCG, CTG,TAG,TCG,TTG\},\\ X_3= & {} \{AAT,AC T,AGT,CAT,CCT, CGT,GAT,GCT,GGT\},\\ X_4= & {} \{ACC,ACG,ACT,AGC,AGG,AGT,ATC,ATG,ATT\},\\ X_5= & {} \{CAA,CAG,CAT,CGA,CGG,CGT, CT A,CTG,CTT\},\\ X_6= & {} \{CCA,CGA,CTA,GCA,GG A,GTA,TCA,TGA,TTA\},\\ X_7= & {} \{GAA,GAC,GAT,GC A,GCC, GCT,GT A,GTC,GT T\},\\ X_8= & {} \{TAA,TAC,TAG,TCA,TCC, TCG,TGA,TGC,TGG\}. \end{aligned}$$

Each of these codes is invariant under the permutation $\pi _{(CG)}$ and $\pi _{(AT)}$ from $L_2$. Thus, there are two equivalence classes induced by the action of $L_2$, namely

$$\begin{aligned} {[}X_8]=\{X_4, X_5, X_7, X_8\} \quad {\text { and }} \quad [X_1]=\{X_1,X_2,X_3,X_6 \} \end{aligned}$$

The following theorem gets to the bottom of the problem and shows that for any word and alphabet cardinality for the classes of maximal (self-complementary) strong comma-free codes, some $L_n$-induced equivalence classes are truly smaller than the order of $L_n$. The result is at most general, since it applies to any *l*-letter ($l\ge 1$) words.

#### Theorem 4.4

*Let* $\Sigma$ *be a finite alphabet with* $|\Sigma |=2n$ *for some* $1< n\in {{\mathbb {N}}}$ *and let* $L_n\subset S_{\Sigma }$ *be the centralizer of some involution* *c* *without fix points. Moreover, let* $l\in {{\mathbb {N}}}$. *Then, the action of* $L_n$ *on* ${\mathcal {C}}$ *induces some equivalence classes of size strictly less than* $|L_n|$ *where* ${\mathcal {C}}$ *is one of the following classes of codes*:

1. *The class of all maximal (=maximum) strong comma-free* *l*-*letter codes*;
2. *The class of all maximal (=maximum)* *c*-*self-complementary strong comma-free* *l*-*letter codes for* $n\ge 3$.

#### Proof

Let $\Sigma$ and *c* as well as $L_n$ and *l* be given as stated in the theorem. We start with the proof of (1), so let ${\mathcal {C}}$ be the class of all maximal strong comma-free *l*-letter codes over $\Sigma$.

1. For $l=1$, there is only one maximal strong comma-free 1-letter code, namely $X=\Sigma$ and, thus, its equivalence class consists of one element but $|L_n| > 1$.
  Let us now consider the case $l=2$. In Fimmel et al. (2017b), it is shown that the number of maximal (=maximum) strong comma-free diletter codes over $\Sigma$ is equal to ${2n\atopwithdelims ()n}=\frac{(2n)!}{(n!)^2}$. This number cannot be divided by the order of the group $|L_n|=n!2^n$, hence there must be an equivalence class of size strictly smaller than $|L_n|$.
  Let us consider now the case $l\ge 3$:
  By (Fimmel et al. 2019, Theorem 5.1.), there is a bijection between the class ${\mathcal {C}}$ and the collection ${\mathcal {P}}$ of all sequences $((T^-_i,T^+_i))_{1 \le i \le l}$ where
  - $(T^-_1,T^+_1)$ is a partition of $\Sigma$ into two non-empty parts; and
  - $(T^-_i,T^+_i)$ is a partition of $\sum \nolimits _{j=1}^{i-1}T^-_jT^+_{i-j}$ for every $i \in \{2, \cdots , l\}$.
  Note that partition means in particular that the sets $T^-_i$ and $T^+_i$ are disjoint and also note that in (a), it is required that $T^-_1$ and $T^+_1$ are both non-empty while $T^-_i$ and $T^+_i$ for $i > 1$ can be empty.
  The bijection above is given by sending such a partition sequence $((T^-_i,T^+_i))_{1 \le i \le l}$ to the code
  $$\begin{aligned} X=\sum \limits _{i=1}^{l-1}T^-_iT^+_{l-i} \end{aligned}$$
  We now fix a permutation $\pi \in L_n$ that has order not equal to 2*n* (e.g. the permutation *c* - note that $n >1$ and hence $2n > 2$). We aim to construct a code in ${\mathcal {C}}$ such that $\pi (X)=X$ and hence the induced equivalence class under $L_n$ would be of non-maximal size. Since the order of $\pi$ is smaller than 2*n*, we can choose $T^-_1$ and $T^+_1$ non-empty and disjoint such that $T^-_1 \cup T^+_1=\Sigma$ and $\pi (T^-_1)=T^-_1$ while $\pi (T^+_1)=T^+_1$. By induction on *i*, we claim that we can also choose $T^-_i$ and $T^+_i$ such that (a) and (b) hold and also $\pi (T^-_i)=T^-_i$ as well as $\pi (T^+_i)=T^+_i$ for all $i \le l-1$. The case $i=1$ is already done. Thus, assume that $\pi (T^-_j)=T^-_j$ and $\pi (T^+_j)=T^+_j$ for all $j < i$. Consequently, also $\pi (T^-_jT^+_{i-j})=T^-_jT^+_{i-j}$ for all $j<i$ and therefore also
  $$\begin{aligned} \pi \left( \sum \limits _{j=1}^{i-1}T^-_jT^+_{i-j}\right) =\sum \limits _{j=1}^{i-1}T^-_jT^+_{i-j} \end{aligned}$$
  It is now obvious that we can choose $T^-_i\not =\emptyset$ and $T^+_i\not =\emptyset$ such that $T^-_i \cap T^+_i=\emptyset$ and $T^-_i \cup T^+_i = \sum \nolimits _{j=1}^{i-1}T^-_jT^+_{i-j}$ with $\pi (T^-_i)=T^-_i$ and $\pi (T^+_i)=T^+_i$. For example, take any element *x* from $\sum \nolimits _{j=1}^{i-1}T^-_jT^+_{i-j}$ and choose $T^-_i$ to be the orbit under $\pi$, i.e. $T^-_i=\{\pi ^k(x) : k \ge 0 \}$. Since $T^-_1$ and $T^+_1$ were both non-empty, it follows that $T^-_i$ does not cover all of $\sum \nolimits _{j=1}^{i-1}T^-_jT^+_{i-j}$ and hence $T^+_i=\sum \nolimits _{j=1}^{i-1}T^-_jT^+_{i-j} \backslash T^-_i$ will do the job.
  It follows that $X=\sum \nolimits _{i=1}^{l-1}T^-_iT^+_{l-i}$ then also satisfies $\pi (X)=X$ and so we have proved (1).
2. We now prove (2). Assume now that ${\mathcal {C}}$ is the class of all maximal (=maximum) *c*-self-complementary strong comma-free *l*-letter codes.
  For $l=1$, there is only one maximal *c*-self-complementary strong comma-free 1-letter code, namely $X=\Sigma$ and, thus, its equivalence class consists of one element but $|L_n| >1$.
  Let us now consider the case $l=2$. In Lemma 4.2, it is shown that the number of maximal *c*-self-complementary strong comma-free diletter codes over $\Sigma$ is equal to $2^n$. This number cannot be divided by the order of the group $|L_n|=n!2^n$, hence there must be an equivalence class of size strictly smaller than that of $L_n$.
  Let us consider now the case $l\ge 3$:
  Again we fix $\pi \in L_n$, so recall that $c \circ \pi =\pi \circ c$. However, this time we require the following extra condition:
  $$\begin{aligned} (+) \quad \quad \pi ^s(N_1N_2) \not =\overset{\longleftarrow }{c(N_1N_2)} \end{aligned}$$
  for all $N_1,N_2 \in \Sigma$, $s\ge 1$ (note that $N_1=N_2$ is not excluded!).
  For instance, if $\Sigma =\{a_i, b_i \mid c(a_i)=b_i, i \le n\}$, then we can choose $\pi =\pi _{(a_1 a_2 \cdots a_n)(b_1b_2 \cdots b_n)}$. Clearly, this $\pi$ commutes with *c* and hence $\pi \in L_n$. Moreover, $\pi$ has order at least 3 since $n\ge 3$ and thus the above condition holds. We now claim that also
  $$\begin{aligned} (++) \quad \pi ^s(N_1N_2 \cdots N_r) \not =\overset{\longleftarrow }{c(N_1N_2 \cdots N_r)} \end{aligned}$$
  for all $N_1,N_2, \cdots , N_r \in \Sigma$ and $s < ord(\pi )$. However, this is immediate since for *r* odd equality above would imply that $\pi ^s(N_{\frac{r+1}{2}})=c(N_{\frac{r+1}{2}})$ and that was excluded by assumption $(+)$. Moreover, if *r* is even, then equality would imply that $\pi ^s(N_{\frac{r}{2}}N_{\frac{r}{2}+1})=c(N_{\frac{r}{2}+1}N_{\frac{r}{2}})$ contradicting $(+)$.
  We proceed as in the proof of (1) in order to construct a code $X \in {\mathcal {C}}$ such that $\pi (X)=X$. Thus, we need to ensure that *X* is also *c*-self-complementary. Therefore, we choose $T^-_1$ and $T^+_1$ as above but in addition we require that $\overset{\longleftarrow }{c(T^-_1})=T^+_1$ (and consequently also $\overset{\longleftarrow }{c(T^+_1})=T^-_1$). For instance, we choose a letter $x \in \Sigma$ and take the orbit *Orb*(*x*) under $\pi$. Then, $\pi (Orb(x))=Orb(x)$. Moreover,
  $$\begin{aligned} \pi (\overset{\longleftarrow }{c(Orb(x))})=\overset{\longleftarrow }{c(\pi (Orb(x)))}=\overset{\longleftarrow }{c(Orb(x))} \end{aligned}$$
  since $\pi$ commutes with *c*. So also $\overset{\longleftarrow }{c(Orb(x))}$ is invariant under $\pi$. We now have to ensure that
  $$\begin{aligned} Orb(x) \cap \overset{\longleftarrow }{c(Orb(x))} = \emptyset . \end{aligned}$$
  But this follows from our property $(++)$. It now follows that
  $$\begin{aligned} \Sigma =Orb(x) \cup \overset{\longleftarrow }{c(Orb(x))} \cup K \end{aligned}$$
  is a disjoint union where $K=\Sigma \backslash (Orb(x) \cup \overset{\longleftarrow }{c(Orb(x))})$and we can just split the rest *K* into two disjoint parts $K_1$ and $K_2$ such that $\overset{\longleftarrow }{c(K_1)}=K_2$ and both are invariant under $\pi$ and finally put $L_1=Orb(x) \cup K_1$ and $R_1=\overset{\longleftarrow }{c(Orb(x))} \cup K_2$. An easy induction argument shows that we can continue this way as in the proof of (1) but each time excluding the self-complementary *r*-letter words and end with a code $X \in {\mathcal {C}}$ that satisfies both conditions $\overset{\longleftarrow }{c(X)}=X$ and $\pi (X)=X$.

$$\square$$

We would like to remark that the above proof certainly also works for $\pi \in S_{\Sigma }$ of order less than 2*n* in case (1). It is not clear if not all of the codes are invariant under some permutation.

#### Remark 4.5

For $n=2$, part (2) of Theorem 4.4 above is not correct as the following example shows. The construction in Theorem 4.4 leads to eight different maximal (=maximum) *c*-self-complementary strong comma-free triletter codes:

$$\begin{aligned} X_1&=\{ TCA, CCA, TGG, TGA\},\\ X_2&=\{ TTG, CTG, CAG, CAA\},\\ X_3&=\{ TGA, GGA, TCC, TCA\},\\ X_4&=\{TTC, GTC, GAC, GAA \},\\ X_5&=\{ AGT, GGT, ACT, ACC\},\\ X_6&=\{ AAC, GAC, GTT, GTC\},\\ X_7&=\{ CAG, AAG, CTT, CTG\},\\ X_8&=\{ CCT, ACT, AGT,AGG\}, \end{aligned}$$

which constitute a single equivalence class of maximal size 8.

$$\begin{aligned} {[}X_1]=\{X_1, X_2, X_3, X_4, X_5, X_6, X_7, X_8\}. \end{aligned}$$

The reason is that in the proof of Theorem 4.4 part 2, it is essential to assume that $n \ge 3$ because for $n=2$ it is not possible to choose $\pi$ with property $(+)$: For instance, $\pi _{(AG)(CT)}$ would imply that $\pi (AC)=GT=\overset{\leftarrow }{c(AC)}$.

The following example shows how the construction from Theorem 4.4 works.

#### Example 4.6

We give an example how the construction in Theorem 4.4 works in case (2). Terminology refers to the proof of Theorem 4.4.

For $\Sigma =\{A,C,G,T,O_1,O_2\}$ and $c=(AT)(CG)(O_1O_2)$, the construction in Theorem 4.4 part 2 gives the following sets when choosing $\pi =\pi _{(ACO_1)(TGO_2)}$.

- $$T^-_1=\{A,C,O_1\}$$, $$T^+_1=\{T,G,O_2\}$$
- $$T^-_1T^+_1=\{AT,CT,O_1T,AG,CG,O_1G,AO_2,CO_2,O_1O_2\}$$ and, hence,
  $$T^-_1T^+_1\backslash \{AT,CG,O_1O_2\}=\{CT,O_1T,AG,O_1G,AO_2,CO_2 \}$$
- $$T^-_2=\{CT,O_1G,AO_2\}$$ and $$T^+_2=\{O_1T,AG,CO_2\}$$.

Finally, one puts

$$\begin{aligned} X= & {} T^-_1T^+_2 + T^-_2T^+_1 = \\&{} \{ AO_1T, AAG, ACO_2, CO_1T, CAG, CCO_2, O_1O_1T, O_1AG, O_1CO_2,\\& CTT, CTG, CTO_2, O_1GT, O_1GG, O_1GO_2, AO_2T, AO_2G, AO_2O_2 \}. \end{aligned}$$

This code *X* is a strong comma-free code which is *c*-self-complementary and invariant under $\pi$.

However, the result of Theorem 4.4 does not mean that all equivalence classes are smaller than $|L_n|$. There can also be equivalence classes of maximal cardinality as the following example shows:

#### Example 4.7

For $\Sigma =\{A,C,G,T,O_1,O_2\}$ and $c=(AT)(CG)(O_1O_2)$, the size of a maximal strong self complementary comma-free triletter code over $\Sigma$ is equal to 18. Let

$$\begin{aligned} X= & {} \{AGT, AGG, AO_1T, AO_1O_1, AGO_1, AO_1G, AO_2G, CO_1G, ACT, \\&CCT, AO_2T, O_2O_2T, O_2CT, CO_2T, CO_1T, CO_2G, CO_1O_1, O_2O_2G\}. \end{aligned}$$

Then, *X* is not invariant under any permutation from $L_3$ and hence *X* generates an equivalence class of size equal to the size of $L_3$.

We shortly give an argument why the code in the above Example is not invariant under any permutation from $L_3$: Clearly, the size of *X* is 18 and it is strong comma-free since all triletters in *X* start with either *A*, *C* or $O_2$ and ends with either *G*, *T* or $O_1$, so first letters and last letters are disjoint. Moreover, *A* appears exactly nine times in the first position, *C* exactly six times and $O_2$ exactly three times. Thus, any permutation from $L_3$ that leaves *X* invariant must fix these three letters and a similar argument on the last letters shows that it must also fix *G*, *T* and $O_1$, hence it is the identity.

#### Remark 4.8

In case of maximal (*c*-self-complementary) strong comma-free triletter codes, the negative result can also be obtained more easily. The numbers of maximum (*c*-self-complementary) strong comma-free triletter codes calculated in Fimmel et al. (2019)

$$\begin{aligned} \begin{array}{lccc}N(2n,3)= \sum \limits _{m=1}^{2n-1}\genfrac(){0.0pt}1{2n}{m}2^{m(2n-m)}, &{} {\text {where}} &{} m=\quad [\frac{n}{2} ]&{} {\text {and}}\\ N(2n,3)= 2^{\tfrac{(n+1)n}{2}},&{} n\ge 3&{} &{}\\ \end{array}, \end{aligned}$$

correspondingly, cannot be divided by the order of the group $|L_n|=n!2^n$.

### Equivalence classes of comma-free codes

We now consider the class of comma-free codes and start with a first observation.

#### Proposition 4.9

*Let* $\Sigma$ *be a finite alphabet with* $|\Sigma |=2n$ *for some* $n\in {{\mathbb {N}}}$ *and let* $L_n\subset S_{\Sigma }$ *be the centralizer of some involution* *c* *without fix points. Moreover, let* ${\mathcal {C}}$ *be the class of*

1. *Maximum diletter comma-free codes over* $\Sigma$;
2. *Maximum* *c*-*self-complementary comma-free triletter codes over* $\Sigma$ *with* $n \not =1$;

*Then, the action of* $L_n$ *on* ${\mathcal {C}}$ *induces some equivalence classes of size strictly less than* $\mid L_n \mid$.

#### Proof

In order to show that $L_n$ induces equivalence classes of sizes $|L_n |$ when acting on the mentioned class ${\mathcal {C}}$ of codes, we show that the size of $L_n$ is not a divisor of the number of such codes.

1. The number of maximal comma-free diletter codes over $\Sigma$ is
  $$\begin{aligned} N(2n,2)=\genfrac(){0.0pt}1{3}{r} \dfrac{(2n)!}{(m!)^3(m+1)^r}. \end{aligned}$$
  where $m:= \left[\frac{2n}{3} \right]$ and $r=2n-3m$ (see Fimmel et al. 2019). Since the order of $L_n$ is $2^n n!$, we expect that the size of an induced equivalence class is
  $$\begin{aligned} \dfrac{\genfrac(){0.0pt}1{3}{r} \dfrac{(2n)!}{(m!)^3(m+1)^r}}{2^n n!}=\dfrac{\genfrac(){0.0pt}1{3}{r}(1 \times 3 \times \cdots \times (2n-1))}{(m!)^3(m+1)^r}. \end{aligned}$$
  However, this number is not an integer. Hence, there must be equivalence classes of smaller size than the size of $L_n$ under the action of $L_n$ on maximal comma-free diletter codes.
2. The number of maximal *c*-self-complementary comma-free triletter codes over $\Sigma$ is 2 for $n=1$, 4 for $n=2$ and 54 for $n=3$. Since in these cases, the order of $L_1$, $L_2$ and $L_3$ is 2, 8 and 48, respectively, the order of $L_n$ is not a divisor of the number of codes except for $n=1$.
  For $n \ge 4$, we expect the size of an equivalence class to be
  $$\begin{aligned} \dfrac{6^{n-1}n!}{2^n n!}=\dfrac{3^{n-1}}{2}. \end{aligned}$$
  However, this is obviously not an integer. Thus, there must be equivalence classes of smaller size than the size of $L_n$ under the action of $L_n$ on maximum self complementary comma-free triletter codes.

$$\square$$

We illustrate the above Proposition by some example where it is also shown that not all equivalence classes need to be of smaller size than the size of $L_n$ in the situation of Proposition 4.9.

#### Example 4.10

The following examples illustrate Proposition 4.9.

1. For $n =2$, the action of $L_2$ induces equivalence classes of different sizes 8 and 4 on the class of maximum diletter comma-free codes over the genetic alphabet ${\mathcal {B}}=\{A,C,G,T\}$.
  - For instance, for
    $$\begin{aligned} X_1=\{AC, AG, AT,CT, GT\} \end{aligned}$$
    the size of its equivalence class under the action of $L_2$ is 4, because $\pi _{(CG)}(X_1)=X_1$.
  - For
    $$\begin{aligned} X_2=\{AC, AG, AT, CT, CG\}, \end{aligned}$$
    the size of its equivalence class under the action of $L_2$ is 8.
2. For $n=3$, the action of $L_3$ induces equivalence classes of different sizes 6, 12 and 48 (the size of $L_3$) on the class of diletter comma-free codes over the alphabet $\Sigma =\{A,C,G,T,O_1,O_2\}$.
  - For
    $$\begin{aligned} X_1= & {} \{AC, AT, AG, AO_1, O_2C, O_2T, O_2G,O_2O_1, CT, CO_1, GT, GO_1\}, \end{aligned}$$
    the size of its equivalence class under the action of $L_3$ is 12.
  - For
    $$\begin{aligned} X_2= & {} \{AC, AG, AO_1, AO_2, TC, TG, TO_1, TO_2, CO_1, CO_2, GO_1, GO_2\} ,\end{aligned}$$
    the size of its equivalence class under the action of $L_3$ is 6.
  - For
    $$\begin{aligned} X_3= & {} \{AC, AT, AG, AO_1, O_2C, O_2T,O_2G, O_2O_1, CG, CO_1, TG, TO_1\},\end{aligned}$$
    the size of its equivalence class under the action of $L_3$ is 48.
3. There are exactly four maximum *c*-self complementary comma-free triletter codes of size 16 over the genetic alphabet ${\mathcal {B}}=\{A,C,G,T\}$ where $c=\pi _{(AT)(CG)}$:
  $$\begin{aligned} X_1= & {} \{AAC, AAT, ACC, ACT, AGC, AGT, ATC, ATT, \\& GAC, GAT, GCC, GCT, GGC, GGT, GTC, GTT\},\\ X_2= & {} \{CAA, TAA, CCA, TCA, CGA, TGA, CTA, TTA,\\& CAG, TAG, CCG, TCG, CGG, TGG, CTG, TTG \},\\ X_3= & {} \{TAC, TAA, TCC, TCA, TGC, TGA, TTC,TTA,\\& GAC, GAA, GCC, GCA, GGC, GGA, GTC, GTA\},\\ X_4= & {} \{AAG, AAT, ACG, ACT, AGG, AGT, ATG, ATT, \\&CAG, CAT, CCG, CCT, CGG, CGT, CTG, CTT\}. \end{aligned}$$
  The equivalence classes of $X_1, X_2, X_3$ and $X_4$ are identical and coincide with the set $\{X_1, X_2, X_3, X_4\}.$

In the above Proposition 4.9, we have seen that in some cases, the equivalence class sizes can be smaller than $|L_n|$ because the number of codes in the code class ${\mathcal {C}}$ is not divisible by the order of the group $L_n$. This is not the case for the maximum triletter comma-free codes:

Their number (compare Fimmel et al. 2019)

$$\begin{aligned} N(2n,3)= \left[ \frac{(1+\sqrt{2})^{2n}}{2}\right] (2n)! \end{aligned}$$

is divisible by the order of the group $L_n$. However, even in this case, there are equivalence classes that are smaller than the order of the group $L_n$. To show this, we first look at the criterion for the maximum triletter comma-free codes proven in Golomb et al. (1958b):

#### Theorem 4.11

(Golomb, Welsh 1958) *Let* $\Sigma$ *be a finite alphabet with* $|\Sigma |=m$, $X\subset \Sigma ^3$ *a triletter code over* $\Sigma$ *with* $|X|=\frac{(m^3-m)}{3}$. *For* $m>2$, *the necessary and sufficient condition that* *X* *constitute a maximum comma- free triletter code is that no initial diletter ever occurs as a final diletter*.

For $m=2$, the Theorem above is not true, as the following example shows (compare Golomb et al. 1958b):

#### Example 4.12

The code $X=\{110, 100\}$ is a maximum comma-free triletter code over $\Sigma =\{0,1\}$ , although 10 occurs both initially and finally.

In the following, we will only need the sufficient condition of Theorem 4.11 , which we will prove for the convenience of the reader:

#### Lemma 4.13

*Let* $\Sigma$ *be a finite alphabet with* $|\Sigma |=m$, $X\subset \Sigma ^3$ *a triletter code over* $\Sigma$ *in that no initial diletter ever occurs as a final diletter. Then,* *X* *is comma-free*.

#### Proof

Let $N_1N_2N_3, \quad N_4N_5N_6\in X, N_i\in \Sigma$. Consider a concatenation

$$\begin{aligned} N_1N_2N_3N_4N_5N_6. \end{aligned}$$

It is clear that $N_2N_3N_4\notin X$ and $N_3N_4N_5\notin X$ since there are no words in *X* beginning with $N_2N_3$ or ending with $N_4N_5$. Thus, *X* is comma-free. $\square$

#### Theorem 4.14

*Let* $S_\Sigma$ *be the symmetric group acting on the elements of the alphabet* $\Sigma$ *with* $|\Sigma |=2n, n\in {{\mathbb {N}}}$, $c\in S_\Sigma$ *an involutory bijection without fix points. Then, there is a maximum triletter comma-free code* $X\subset \Sigma ^3$ *with*

$$\begin{aligned} c(X)=X. \end{aligned}$$

#### Proof

Without loss of generality, we may assume that

$$\begin{aligned}&\Sigma =\{1,2,\ldots ,2n\}\quad {\text{ and }}\quad c=(12)(34)(56)\ldots ((2n-1)2n), i.e\\&c:\Sigma \rightarrow \Sigma , \quad c(x)= {\left\{ \begin{array}{ll} x+1,&{} x\quad {\text {is odd}}\\ x-1,&{} x {\quad {\text {is even}}}\\ \end{array}\right. } \end{aligned}$$

Further let $N_1N_2N_3\in \Sigma ^3\setminus \{xxx|x\in \Sigma \}$, $[N_1N_2N_3]$ its (complete) cyclic equivalence class and

$$\begin{aligned} M:=\max \{N_1, N_2, N_3\},\quad m:=\min \{N_1, N_2, N_3\}. \end{aligned}$$

Let us remark that $M>m$ is always valid.

**1. Case**: *m* *is even or* *m* *is odd and* $(m+1) \notin \{N_1, N_2, N_3\}$.

In this case, we choose

$$\begin{aligned} xyz\in [N_1N_2N_3] \end{aligned}$$

with $y=m, x\ge y=m, z>y=m$. This choice is possible because of the definition of *m* and because not all three positions $N_i$ are equal. Let us remark that obviously in this case, no initial diletter ever occurs as a final diletter.

Then, $c(xyz)=x'y'z'$ has the same shape again, i.e. $x'\ge y', z'> y'$.

**2. Case**: *m* *is odd and* $(m+1)\in \{N_1, N_2, N_3\}$
**2.1**: *There are* $$i,j\in \{1,2,3\}, i\ne j$$ $$N_i=N_j$$.

In this case, we choose

$$\begin{aligned} xyz\in [N_1N_2N_3] \end{aligned}$$

with $$y=x, z\ne x, |y-z|=1$$. Thus,

$$\begin{aligned} c(xxz)=zzx \end{aligned}$$

has the same shape again and no initial diletter ever occurs as a final diletter in both cases 1. and 2.1.

**2.2**: *For all* $$i,j\in \{1,2,3\}, i\ne j$$ $$N_i\ne N_j$$

In this case, we choose

$$\begin{aligned} xyz\in [N_1N_2N_3] \end{aligned}$$

with $x=M>m+1$. Then, $c(x)>m+1$ since $m+1$ is even and $c(y)=z$. Thus, *c*(*xyz*) has the same shape again and no initial diletter ever occurs as a final diletter.

The so constructed code $X\subset \Sigma ^3\setminus \{xxx|x\in \Sigma \}$ is comma-free according to Theorem 4.13:

Let’ us take a look at $xyz\in X$. Due to the construction, we always have $x\ge y$ if $y\ne z$ applies. Therefore, an initial diletter *xy* with $x=y$ can never appear as a final diletter. If $x>y$ applies, *xy* cannot appear as final diletter in every tuple from the first case, because $y<z$ applies to it. In case 2. the final diletter always looks like $(m+1)m$ or $m (m+1)$ with an odd *m*. The constellation $(m+1)m$ can only appear as the initial diletter in case 1, with an even *m*. In summary, in the constructed code, an initial diletter can never appear as the final diletter, so the code is comma-free.

*X* is also maximum (in fact $\mu$-maximum), because we have chosen exactly one element from each complete equivalence class. Furthermore, according to construction, $c(X)=X$ is valid.$\square$

Again for the convenience of the reader, we illustrate the above Theorem by an example.

#### Example 4.15

Let ${\mathcal {B}}=\{A,C,G,T \}$ be the genetic alphabet. If we assign to *A* 1 , to *T* 2, to *C* 3 and to *G* 4, the known complementarity transformation will correspond to the *c* defined in the Theorem 4.14. The code *X* we define according to the theorem above will look like this:

$$\begin{aligned} X= & {} \{AAC, AAG, AAT, CAC, GAC, CTA, CAG, GAG, GTA, CAT, \\&GAT, TTA, CCG, CTC, GGC, GTC, CTG, TTC, GTG, TTG \}. \end{aligned}$$

The code is maximum comma-free and invariant under *c*.

As an immediate corollary we obtain

#### Corollary 4.16

*Let* $\Sigma$ *be a finite alphabet with* $|\Sigma |=2n$ *for some* $n\in {{\mathbb {N}}}$ *and let* $L_n\subset S_{\Sigma }$ *be the centralizer of some involution* *c* *without fix points. Moreover, let* ${\mathcal {C}}$ *be the class of maximum triletter comma-free codes over* $\Sigma$. *Then, the action of* $L_n$ *on* ${\mathcal {C}}$ *induces some equivalence classes of size strictly less than* $|L_n|$.

#### Proof

Immediately follows from Theorem 4.14. $\square$

Again we illustrate the above Corollary by some example.

#### Example 4.17

For the genetic alphabet ${\mathcal {B}}=\{A,C,G,T\}$ the action of $L_2$ induces equivalence classes of different sizes 8, 4 and 2 on the class of maximum triletter comma-free codes:

1. For instance, for
  $$\begin{aligned} X_1= & {} \{AAC, AAG, AAT, CAC, CAG, CAT, CCT, CGC, CGT, GAC, \\&GAG, GAT, GGC, GGT, TAC, TAG, TAT, TCT, TGC, TGT \} \end{aligned}$$
  the size of its equivalence class under the action of $L_2$ is 8.
2. For
  $$\begin{aligned} X_2= & {} \{ATC, ATG, ATT, CAA, CAC, CAG, CGC, CGG, CTC, CTG, \\&CTT, GAA, GAC, GAG, GTC, GTG, GTT, TAA, TAC, TAG \} \end{aligned}$$
  the size of its equivalence class under the action of $L_2$ is 4.
3. For
  $$\begin{aligned} X_3= & {} \{AAC, AAG, AAT, CAC, CAG, CAT, CGG, CTA, CTC, CTG,\\& GAC, GAG, GAT, GCC, GTA, GTC, GTG, TTA, TTC, TTG \} \end{aligned}$$
  the size of its equivalence class under the action of $L_2$ is 2.

### Equivalence classes of circular and $C^l$- codes

As explained above, the task of the article is motivated by the successful division of the set of all maximum self-complementary $C^3$-codes and the associated practical benefit for the research of code classes. So it was an obvious idea to try to do the same with the class of maximum self-complementary circular codes. This failed (see Lemegne 2015). For instance, dropping the $C^3$-property it turned out that for the class of maximal *c*-self-complementary circular codes, the action of $L_2$ induces 64 equivalence classes of size 8 but also 2 equivalence classes of size 4:

#### Example 4.18

Let $B=\{A,C,G,T\}$ be the genetic alphabet. Then, the following two maximum *c*-self-complementary circular codes generate equivalence classes of size 4 under the action of $L_2$:

$$\begin{aligned} X_1= & {} \{AAC, AAG,AAT,ACC,GAC,ACT, AGC,GGA,AGT,ATC,\\&GAT, ATT, GCC, TCC, GGC, GTC, GCT, CTT, GGT, GTT\}, \end{aligned}$$

and

$$\begin{aligned} X_2= & {} \{AAC, GAA,AAT,ACC,GAC,ACT, AGC,AGG,AGT,ATC,\\& GAT, ATT, GCC, CCT, GGC, GTC, GCT, TTC, GGT, GTT\}. \end{aligned}$$

The reason for the smaller size of the equivalence class is that

$$\begin{aligned} \pi _{(TC)}(X_1)=\pi _{(AG)}(X_1)=X_1, \pi _{(TC)}(X_2)=\pi _{(AG)}(X_2)=X_2. \end{aligned}$$

For the class of maximum dinucleotide circular codes the division into equally sized equivalence classes under the action of $L_n$ then works again:

#### Lemma 4.19

*Let* $\Sigma$ *be a finite alphabet with* $|\Sigma |=2n$ *for some* $n\in {{\mathbb {N}}}$ *and let* $L_n\subset S_{\Sigma }$ *be the centralizer of some involution* *c* *without fixed points. Let* ${\mathcal {C}}$ *be the class of all maximal diletter circular codes. Then, the action of* $L_n$ *induces equally sized equivalence classes (of size* $\mid L_n \mid$) *on* ${\mathcal {C}}$.

#### Proof

To show that all equivalence classes indeed have the same size we recall a result from Fimmel et.al in Fimmel et al. (2019) where it was proved that any maximal diletter circular code has a presentation of the following form:

$$\begin{aligned} X=\{N_iN_j| i,j=1,2,\ldots ,2n, i<j, N_i, N_j\in \Sigma , N_i\ne N_j\} \end{aligned}$$

where $\Sigma =\{N_1, \ldots , N_{2n}\}$. Consequently, the first diletter $N_1$ appears $2n-1$ times as a prefix of words from *X*, the second letter $N_2$ appears $2n-2$ times and so on. The last but one letter $N_{2n-1}$ has only one occurrence as a prefix of some word from *X* while the last letter $N_{2n}$ never occurs as a prefix. Now, assuming that $\pi \in L_n$ satisfies $\pi (X)=X$, we conclude that for every $i \le 2n$, we must have $\pi (N_i)=N_i$ which means that $\pi =id.$ Thus, no maximal diletter circular code is invariant under a nontrivial $\pi \in L_n$. $\square$

With the theorem below, we try to explain which code properties are responsible for the success or failure of a code class division into classes of equal size. Recall that $\mu$-maximum means that the code contains exactly one *l*-letter from each complete equivalence class.

#### Theorem 4.20

*Let* $\Sigma$ *be a finite alphabet or arbitrary size* *m* *and let* ${\mathcal {C}}$ *be the class of all* $\mu$-*maximum* *l*-*letter* $C^{l}$ *codes for some natural number* *l*. *Then, the action of* $S_{\Sigma }$ *induces equally sized equivalence classes on* ${\mathcal {C}}$.

#### Proof

We try to prove the above theorem by showing that for any $X \in {\mathcal {C}}$ and any $\pi \in S_{\Sigma }$ with $\pi \not = id$ we have $\pi (X) \not = X$. First, we collect some facts that we would like to use. So assume $\Sigma$ and $X \in {\mathcal {C}}$ are given, i.e. *X* is a maximum *l*-letter $C^{l}$ code. Last but not least assume that $\pi \in S_{\Sigma }$ such that $\pi (X)=X$. Then, the following hold:

(i) Let $k=ord(\pi )$, i.e. *k* is the smallest natural number *s* such that $\pi ^s=e$, the identity. Then, also $\pi ^{s}(X)=X$ for all $s \le k$. Moreover, $\pi ^{k-1}=\pi ^{-1}$;
(ii) Since $\pi ^s(X)=X$ and $\pi$ obviously commutes with $\alpha _1, \cdots , \alpha _{l-1}$ we also have that $\pi ^s(\alpha _i(X))=\alpha _i(X)$ for all $s \le k$ and $i \le l-1$;
(iii) Any *l*-letter $N_1 \cdots N_l \in \Sigma ^l$ must be contained in either *X* or one of the $\alpha _i(X)$ ($i \le l-1$) by maximality of *X* provided $N_1 \cdots N_l$ generates a complete equivalence class.
(iv) If $x=N_1 \cdots N_l$ is an *l*-letter such that for some *i* we have $N_i=N_{i+1}$ and for all $j \not =i$ we have $N_j \not = N_{j+1}$ (i.e. *x* has only one pair of identical consecutive letters with the convention that $l+1=1$), then *x* generates a complete equivalence class (because any shift of *x* moves the only two identical consecutive letters to another position).

We now write $\pi$ as a direct product of disjoint cycles, i.e.

$$\begin{aligned} \pi = \pi _1 \cdots \pi _l \end{aligned}$$

where each $\pi _j$ is of the form $\pi _{(N_1, \cdots , N_k(j))}$ for different $N_1, \cdots , N_{k(j)} \in \Sigma$. Since the cycles are disjoint, it follows that for each $\pi _j$ also conditions (*i*) to (*iii*) from above hold when considered on $X \cap \{N_1, \cdots , N_{k(j)}\}$. Thus, we assume without loss of generality that $\pi =\pi _1=\pi _{(N_1, \cdots , N_k)}$ is a cycle keeping in mind that from now on all arguments have to use *l*-letter word from $\{N_1, \cdots , N_{k(j)}\}$ only.

We now distinguish cases:

- **Case 1:** *k* is even.

In this case, let $s=\frac{k}{2}$. Then $\pi ^s(N_1)=N_{s+1}$ and $\pi ^s(N_{s+1})=N_1$.

- ${\underline{l\,\mathbf{is\,odd}}}$ Let $x=N_1N_{s+1} \cdots N_1N_{s+1}N_1$. By condition (*iv*), it follows that *x* generates a complete equivalence class. Hence, we may assume without loss of generality that $x \in X$ by condition (*iii*) (note that *x* has exactly two identical consecutive letters $N_1$). However, $\pi ^s(x)=N_{s+1}N_1 \cdots N_{s+1}N_1N_{s+1} \in X$ then implies that  has two decompositions contradicting the circularity of *X*.
- $${\underline{l\,\mathbf{is\,even}}}$$
  Let  where each coloured part consists of exactly $\frac{l}{2}$ letters. Again, by condition (*iv*), the word *x* generates a complete equivalence class (note that it has exactly to identical consecutive letters $N_{s+1}$) and by condition (*iii*), we may assume that $x \in X$. However,  is then in the same equivalence class as *x* contradicting circularity of *X*.

- **Case 2:** *k* is odd.
  We need to distinguish cases again according to the size of *l*.
  - $${\underline{l < k}}$$
    Choose $x=N_1 \cdots N_kN_1 \cdots N_k \cdots N_1 \cdots N_k=(N_1 \cdots N_k)^l$ - the concatenation of *l* copies of $N_1 \cdots N_k$. Since $l < k$, the word *x* is a concatenation of *k* words of length *l*, say $y_1 \cdots y_k$ with $y_1=N_1 \cdots N_l$. Since all $N_i$ were different, $y_1$ generates a complete equivalence class and hence we may assume that $x \in X$ by condition (*iii*). Moreover, $\pi ^l(y_i)=y_{i+1}$ for all $i < k$ and $\pi ^l(y_k)=y_1$. Thus, also $y_2, \cdots , y_k \in X$. This shows that $x \in X^k$. However, a similar argument shows that $\pi$ applied to *x* gives $\alpha _1(x)$ and hence also $\alpha _1(X) \in X^k$ - a contradiction to the circularity of *X*.
  - $${\underline{l\,\ge k\,\mathbf{and}\,l\,\not \equiv \,0\, \mathbf{modulo}\,k}}$$
    In this case, the same construction as in Case *a*) applies and yields a contradiction. Note that also in this case, the $y_1, \ldots , y_k$ generate complete equivalence classes.
  - $${\underline{l\,\ge k\,\mathbf{and}\,l\,\equiv \,0\,\mathbf{modulo}\,k}}$$
    Let $l=mk$ and choose $x=N_1 \ldots N_1 N_2 \ldots N_2 \ldots N_k \cdots N_k$—the concatenation of blocks of *m* copies of $N_i$. Then, clearly *x* generates a complete equivalence class and by (*iii*) we may assume that $x \in X$. However, then $\pi (x)= N_2 \cdots N_2 N_3 \ldots N_3 \ldots N_k \ldots N_k N_1 \ldots N_1 \in X$ contradicts circularity since obviously *x* and $\pi (x)$ are in the same equivalence class.

$$\square$$

A first corollary is immediate.

#### Corollary 4.21

*Let* $\Sigma$ *be a finite alphabet or even size* 2*n* *and let* ${\mathcal {C}}$ *be the class of all* $\mu$-*maximum* *c*-*self-complementary* *l*-*letter* $C^{l}$ *codes for some natural number* *l* *where* $c\in S_{\Sigma }$ *is an involution without fix points. Then, the action of* $L_n$ *induces equally sized equivalence classes on* ${\mathcal {C}}$.

#### Proof

Follows directly from Theorem 4.20. $\square$

We would like to remark that it is an open question if maximum and $\mu$-maximum are the same for $C^l$-codes. However, it is true for circular codes. We have an immediate corollary that is well known.

#### Corollary 4.22

*Let* $\Sigma =\{A,C,G,T\}$ *be the genetic alphabet and* $c=\pi _{(AC)(GT)}$ *as well as* $L_2=C_{S_{\Sigma }}(c)$. *Let* ${\mathcal {C}}$ *be the class of all maximal* *c*-self-*complementary triletter* $C^{3}$ *codes. Then, the action of* $L_2$ *induces equally sized equivalence classes (of size* $\mid L_2 \mid =8$) *on* ${\mathcal {C}}$.

#### Proof

Follows directly from the above Theorem 4.20 since maximum in this case is indeed the same as $\mu$-maximum.$\square$

## Conclusions

In the present work, classes of *l*-letter codes over general alphabets $\Sigma$ have been investigated with respect to their behaviour under the natural action of a specific subgroup *L* of the symmetric group $S_{\Sigma }$ acting on the letters of the alphabet. These codes all share some error-detecting and -correcting properties of decreasing strength from strong comma-freeness to comma-freeness to circularity. The group *L* was motivated from a biological context where the class of maximal circular self-complementary $C^3$-codes had been found in nature and seem to play an important role for frame retrieval during the translation process in the ribosome (see Arquès and Michel 1996; Michel 2015, 2017). Self-complementarity originates from the double helix structure of the DNA but in general it can be seen as a kind of correspondence between letters, e.g. in the binary case 0 and 1 correspond to each other. Based on these findings, several models of the evolution of the genetic code were developed proposing strong comma-free or comma-free ancient predecessor codes of the current standard genetic code (see Fimmel et al. 2020, 2018). Passing from the biological context to coding theory and the field of signal processing all classes of codes were deeply investigated with respect to their error-revealing properties using graph theory and combinatorics (see Ball and Cummings 1976a; Fimmel et al. 2020, 2018, 2019, 2017a, b, 2016, 2014; Levenshtein 2004).

Three important observations had motivated our research. The first one is that codes belonging to the same equivalence class under the action of the group *L* share identical error-detecting and error-correcting properties. Thus, it seemed reasonable to investigate how large such equivalence classes turn out to be. In the genetic code setting, it had already been observed that the 216 maximal self-complementary $C^3$-codes are divided into 27 equivalence classes of size |*L*|. However, for general circular codes or comma-free codes, this was wrong (see Keller 2014; Lemegne 2015). The second motivation was given by several research studies showing that there are variants of the genetic code that are based on six bases and other research studies proposing ancient genetic codes that used dinucleotides, tetra-nucleotides or even penta-nucleotides for coding amino acids (see Demongeot and Seligmann 2020; Fimmel et al. 2020; Malyshev et al. 2014; Michel and Pirillo 2013). Therefore, it was reasonable to study codes not only in the triletter case over the genetic alphabet with four letters but general *l*-letter codes over larger alphabets. The last motivation came from a series of papers by Seligman (see Demongeot and Seligmann 2020, 2019; Michel and Seligmann 2014; Seligman 2016) who discovered so-called Swinger RNA which is RNA that can be obtained from different RNA by applying a systematic change of bases (i.e. by applying one of the transformations from *L*). It was speculated that nature may use this mechanism in order to encode not only one set of information in DNA but 8 (the size of *L*) or even 24 (the size of $S_{\{A,C,G,T\}}$) sets at the same time. These Swinger copies would then use the corresponding circular code in the equivalence class of codes under *L* for frame synchronization.

Our results clarify completely the situation for several classes of codes showing the (non-) existence of equivalence classes of size |*L*| or strictly smaller size. Besides the canonical application to the genetic code or the extended (up to six coding nucleotide bases) genetic code, the case of the binary alphabet is especially important for applications in signal processing. It proves to be a special case for classes of maximal strong comma-free and maximum self-complementary comma-free trinucleotide codes. Namely, only in this case, the corresponding equivalence classes all have the maximum possible size.

Moreover, the results of the present investigation suggest that the code properties responsible for the maximal size of equivalence classes are that the codes are maximally large and retain their error-detecting properties in all frames ($C^l$ property).
